# Proteins and microRNAs are differentially expressed in tear fluid from patients with Alzheimer’s disease

**DOI:** 10.1038/s41598-019-51837-y

**Published:** 2019-10-28

**Authors:** Aidan Kenny, Eva M. Jiménez-Mateos, María Ascensión Zea-Sevilla, Alberto Rábano, Pablo Gili-Manzanaro, Jochen H. M. Prehn, David C. Henshall, Jesús Ávila, Tobias Engel, Félix Hernández

**Affiliations:** 1 Department of Physiology & Medical Physics, Royal College of Surgeons in Ireland, D02YN77 Dublin, Ireland; 2 Discipline of Physiology, School of Medicine, Trinity College Dublin, The University of Dublin, Dublin, Ireland; 3 Centro de Biología Molecular “Severo Ochoa” (CSIC-UAM), c/Nicolás Cabrera 1, Universidad Autónoma de Madrid, Cantoblanco, 28049 Madrid Spain; 4 Department of Neuropathology and Tissue Bank, Fundación CIEN, Instituto de Salud Carlos III, Madrid, Spain; 5 Servicio de Oftalmología, Fundación Hospital Alcorcón, Madrid, Spain; 6 FutureNeuro Research Centre, RCSI, D02YN77, Dublin, Ireland; 7 Centro Investigación Biomédica en Red Enfermedades Neurodegenerativa (CIBERNED), Madrid, Spain

**Keywords:** Alzheimer's disease, Prognostic markers

## Abstract

Alzheimer’s disease (AD) is characterized by a progressive loss of neurons and cognitive functions. Therefore, early diagnosis of AD is critical. The development of practical and non-invasive diagnostic tests for AD remains, however, an unmet need. In the present proof-of-concept study we investigated tear fluid as a novel source of disease-specific protein and microRNA-based biomarkers for AD development using samples from patients with mild cognitive impairment (MCI) and AD. Tear protein content was evaluated via liquid chromatography-mass spectrometry and microRNA content was profiled using a genome-wide high-throughput PCR-based platform. These complementary approaches identified enrichment of specific proteins and microRNAs in tear fluid of AD patients. In particular, we identified elongation initiation factor 4E (eIF4E) as a unique protein present only in AD samples. Total microRNA abundance was found to be higher in tears from AD patients. Among individual microRNAs, microRNA-200b-5p was identified as a potential biomarker for AD with elevated levels present in AD tear fluid samples compared to controls. Our study suggests that tears may be a useful novel source of biomarkers for AD and that the identification and verification of biomarkers within tears may allow for the development of a non-invasive and cost-effective diagnostic test for AD.

## Introduction

Alzheimer’s disease (AD) is an age-related neurodegenerative disorder characterised by wide-spread neuron and synapse loss resulting in phenotypic cognitive deficits and eventual onset of dementia. The histopathologic hallmarks of AD include extracellular plaques containing the amyloid-β (Aβ) peptide and neurofibrillary tangles containing the hyperphosphorylated protein tau^1^. The early pathogenic process of AD is clinically silent, developing over many years before a clinical diagnosis is possible^2^. During the early pathogenesis of AD, one of the few detectable changes is a transition from cognitively normal to a mild cognitive impairment (MCI). MCI is a high risk state for the development of AD but is a heterogeneous condition common among many neurodegenerative diseases as well as occurring in unaffected individuals with a large percentage of cases never converting to dementia making this state very susceptible to misdiagnosis^3^.

The uncertainty in identifying the cognitively unimpaired members of a research cohort independent from biomarker profiles in the earliest stages of AD and reliance on the onset of cognitive loss for diagnosis where molecular pathology of AD has already reached a critical stage is a major limitation in clinical trials where earlier interventional therapies for AD show more promise^4^. Detection of these earlier stages of AD is reliant on neuroimaging biomarkers, based on magnetic resonance imaging (MRI), functional magnetic resonance imaging (fMRI) and computed tomography (CT) scanning techniques^5–8^ as well as protein biomarkers from cerebrospinal fluid (CSF)^9,10^. Neuroimaging and CSF biomarkers both have significant challenges in their practicality, with neuroimaging being severely limited by cost and availability while obtaining CSF biomarkers requires an invasive sampling procedure. This has driven investigation into possible biomarkers present in less invasive body fluids such as blood^11–13^ or urine^14^.

Tears represent a non-invasive biofluid but are a largely unexplored biomarker source. There have been very limited studies on tears as a source of biomarkers for neurological disorders although they are known to contain proteins, nucleotides and other molecules^15–17^. Currently only one study has investigated tears as a source of molecular biomarkers in AD^18^. This lack of investigation into tear fluid is striking due to its close relationship to the eye which has been shown to have strong connections to AD pathology with numerous putative markers for AD based on the effect of AD upon the eye^19^. These include distinct pupillary size changes and changes in the responses to light^20^ as well as plaque formation in retina and lenses of AD patients which illustrates the powerful effect AD pathology has on the various components of the eye^21,22^. Tears have also been identified as a source of biomarkers in other neurological disorder including multiple sclerosis in which tear-based proteins were suggested as a screening test before progressing to more invasive and definitive diagnostic techniques such as CSF-based ELISA^23^.

In the present work we investigated possible differences in tear composition of healthy aged controls and cognitively impaired patients. This was performed by high throughput proteomic and PCR-based platforms to assess protein content and relative expression of microRNAs. MicroRNAs are a class of short, non-coding molecules which function in the post-transcriptional regulation of mRNA and protein levels in the cell^24^. MicroRNAs have become an area of particular interest in AD research, with critical roles in the pathogenesis of the disease reported^25,26^. Due to their biochemical characteristics, including relative stability in extracellular environments and their presence in extracellular particles such as exosomes^27^, microRNAs have attracted much attention as possible biomarkers for brain diseases including AD^28^. Increasing evidence suggest AD-specific concentration changes of microRNAs in various biofluids including plasma and CSF^29,30^. The presence of microRNAs within tear fluid has been reported in healthy humans^31^, however, to date has not been investigated in AD or any other brain disease.

Here, by analysing samples from patients with MCI and AD, we report disease-specific differences in tear content at the protein level as well as changes in microRNA concentrations, thereby providing the proof-of-concept that tear fluid can potentially be used as novel biofluid for the diagnosis of AD.

## Results

### Protein composition changes in tear fluid of AD patients

**Figure 1:**
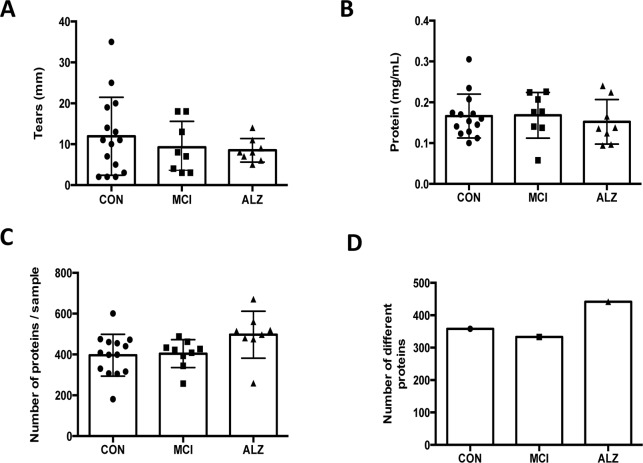
Protein concentration and volume of collected tear fluids. (**A**) Tear secretion volume measured as the length of the wet strip (in mm) (ANOVA, p = 0.531). (**B**) Total protein concentration in 500 μl of PBS with a cocktail of peptidase inhibitors where strips were submerged (p = 0.622). (**C**) Number of different proteins present per sample in tears analysed in this study after analysis by LC-MS/MS (p = 0.067). (**D**) Number of different proteins present in each cohort. CON, control; MCI, Mild cognitive impairment; AD, Alzheimer’s disease. The bars represent mean values with the standard error.

The proteomic content of tear fluid has been previously reported in healthy volunteers as well within other neurological disorders such as multiple sclerosis^23^. Here we explored whether AD pathology led to a distinct proteomic fingerprint in tear fluid which could be used as potential biomarker for AD diagnosis. First, we analysed if secretion of tears was altered. Tear production (flow rate) was 12 ± 9 mm/5 min in controls which was not significantly different in patients with either MCI or AD (9.25 ± 6.3 and 8,5 ± 2,9/5 min respectively, Fig. 1A). Total protein content in tears was also similar between controls and the two patient groups (Fig. 1B).

Next we performed liquid chromatography-mass spectrometry (LC-MS/MS) analysis to establish the possible disease-specific tear protein profile. To ensure quality of our samples, we examined the presence of well-established tear markers such as lysozyme, lipocalin 1, serotransferrin and retinal dehydrogenase 1 (ALDH1A1).

The number of proteins present in each tear sample was then analysed. No significant difference was observed between the average number of proteins present in each condition, although there was a trend toward higher numbers of unique proteins in tears from AD patients (Fig. 1C). To investigate what processes might be over-represented, we conducted a gene ontology analysis using the Database for Annotation, Visualization and Integrated Discovery (DAVID)^32,33^. However, comparing the three groups (controls, MCI and AD) no differences were found.

**Figure 2:**
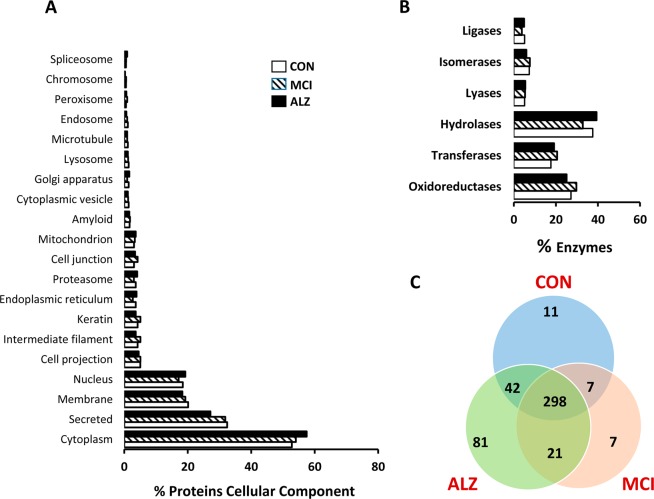
Functional genes and gene groups. (**A**) Gene Ontology (GO) analysis for categories ‘cellular component’ (http://www.uniprot.org/) for proteins present in tear samples. (**B**) Enzymes present in tear samples according to the six main divisions (classes). (**C**) Venn diagram showing which proteins are shared by each cohort. The overlapping region demonstrate that 298 proteins are present in the three tear categories. CON, control; MCI, Mild cognitive impairment; AD, Alzheimer’s disease.

A more detailed analysis of proteins detected in all samples showed that around 40% of identified proteins were present only in one or two samples of each cohort (40% in control, 38% in MCI and 40% in AD). These proteins were eliminated from further analysis and proteins that appeared in at least 50% of samples of each cohort were retained. After filtering, the number of proteins that appeared in at least 50% of the samples of each cohort was 358 in control samples, 333 in MCI samples and 442 in AD samples (Fig. 1D). Surprisingly, peptides from tau protein or amyloid precursor protein (APP) were not detected in any of the samples. Proteins present in control, MCI and AD tears showed a similar proportion of protein distribution among different cellular components (Fig. 2A). In addition, no differences were found in levels of tear-specific enzymes between groups (Fig. 2B).

**Figure 3:**
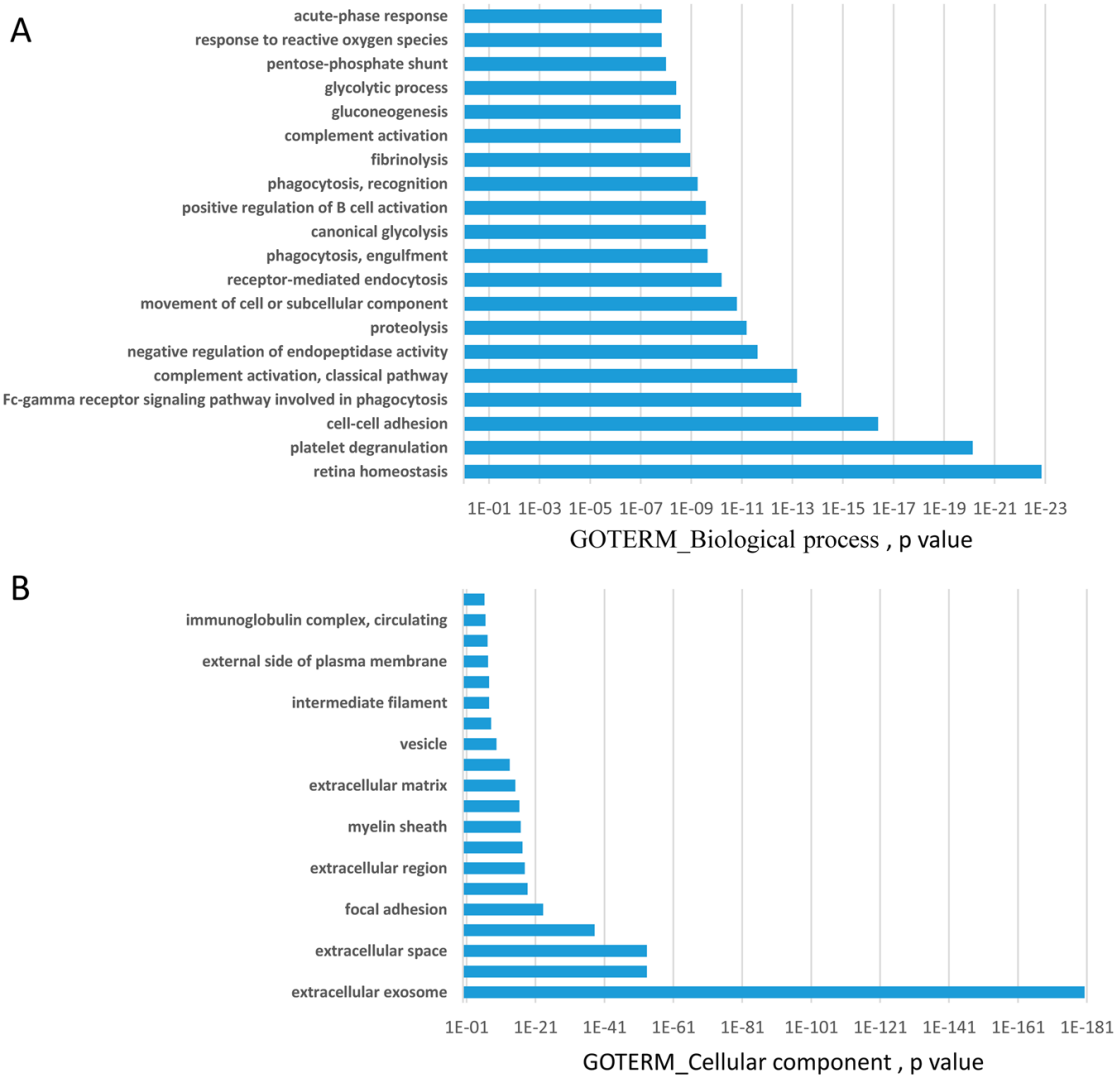
GO term analysis of common proteins present in tear samples. GO term analysis by Biological process (**A**) and by Cellular component (**B**) of those 298 common proteins present in the three tear groups. These proteins were mainly related with retina homeostasis and extracellular exome.

**Figure 4:**
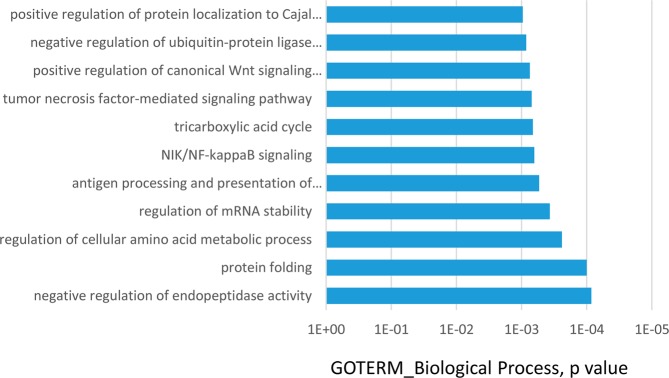
GO term analysis of proteins present in tear samples of AD patients. Proteins present in AD samples did not show a clear GO term pattern (Biological process), being the p values higher than 1 × 10^−4^. The most enriched categories contribute to regulation of endopeptidase activity, protein folding, regulation of cellular amino acid metabolic process and regulation of mRNA stability.

We then analysed how these proteins (present in at least 50% of all samples) were distributed among the three groups. First, we observed that 298 proteins were present in control, MCI or AD samples, while 81 proteins were only found in samples from AD patients and 7 were unique to MCI (Fig. 2C). As before, to analyze what cellular processes might be overrepresented in these proteins, gene ontology analysis was conducted using DAVID. Figure 3A shows that GO term analysis by Biological process of those 298 common proteins were mainly related with retina homeostasis (Fig. 3A, p < 1 × 10^−23^) confirming the majority of proteins in tears are housekeeping proteins. Figure 3B shows GO term analysis by Cellular Component confirming that proteins present in tears fall mainly in the category of “Extracellular Exome”. GO analysis of the 81 proteins present in at least 50% of AD-samples did not identify any enrichment for a specific GO term with no pathways showing p values higher than 1 × 10^−4^ (Fig. 4). The most enriched categories identified contribute to the regulation of endopeptidase activity, protein folding, the regulation of cellular amino-acid metabolic processes and the regulation of mRNA stability (Fig. 4).

### Identification of AD-specific proteins in tear fluid

To identify a potential tear diagnostic protein biomarker of AD, we analysed those proteins present in at least 50% of samples from each group. We identified 12 proteins present in at least five AD samples (55%) and only two control samples (13%) or less (Table 1). Interestingly, most of these proteins show an increase in MCI compared with control samples, suggesting an incremental change in disease progression. Interestingly, a GO term analysis of these 12 proteins revealed that 11 fall into the category of “metabolic processes”. However, how exactly these 12 proteins are mechanistically involved in AD remains unclear.

**Table 1:** Potential AD-specific tear biomarkers proteins.

UNIPROT	CONTROL*	MCI*	AD*	PROTEIN DESCRIPTION	FUNCTION
O43242	2	3	5	26S proteasome non-ATPase regulatory subunit 3	protein homeostasis
O43776	2	1	7	Asparagine--tRNA ligase, cytoplasmic	Protein biosynthesis
P06730	0	2	5	Eukaryotic translation initiation factor 4E	Protein biosynthesis
P07357	1	1	6	Complement component C8 alpha chain	Innate and adaptive immune response by forming pores in the plasma membrane of target cells
P07954	2	1	6	Fumarate hydratase, mitochondrial	part of the pathway tricarboxylic acid cycle
P08185	2	3	5	Corticosteroid-binding globulin	Major transport protein for glucocorticoids and progestins in the blood
P30040	2	1	6	Endoplasmic reticulum resident protein 29	Processing of secretory proteins within the endoplasmic reticulum
P50452	1	3	6	SERPINB8	Serine protease inhibitor and has an important role in epithelial desmosome-mediated cell-cell adhesion
P67936	2	3	8	Tropomyosin alpha-4 chain	Binds to actin filaments
Q03591	2	2	5	Complement factor H-related protein 1	Involved in complement regulation
Q3LXA3	1	2	8	Triokinase/FMN cyclase	Phosphorylation of dihydroxyacetone and of glyceraldehyde, and the splitting of FAD
Q9Y3F4	1	0	5	Serine-threonine kinase receptor-associated protein	Splicing of cellular pre-mRNAs contributing to spliceosomal snRNP biogenesis. TGF-beta signaling

*Number of tear samples in which the protein could be detected.

The most relevant protein is eukaryotic translation initiation factor 4E (eIF4E) which appears in five AD samples (55%) but was absent in all control tear samples. Moreover, Tropomyosin alpha-4 chain and Triokinase/FMN cyclase are present in eight samples out of nine, although they are also present in some control and MCI samples (Table 1). eIF4E was selected for verification as potential biomarker for AD. Thus, we carried out Western blot with anti-eIF4E, however no signal was obtained with any of the tear samples analyzed (Supplementary Fig. 1).

### Increased microRNA concentration in tears from AD patients

**Figure 5:**
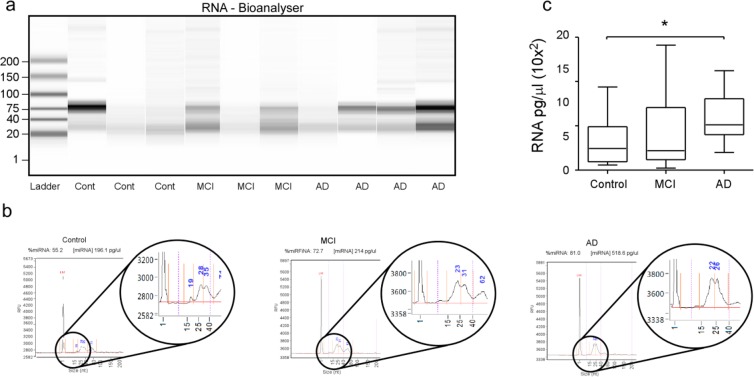
Tear RNA quality analysis and quantification. (**a**,**b**) RNA verification and quantification by AATI fragment analyser. RNA is separated out based on size (nucleotides (nt)) and quantified by signal intensity. (**b**) Quantification of the percentage of small-sized RNA extracted within the microRNA size range (10–40 nt) and the total quantity of microRNA-sized RNA molecules in each sample. (**c**) Comparison of microRNA concentration between conditions, control (n = 22), MCI (n = 16) and AD (n = 12) (p = 0.0189) ANOVA.

Analysis of microRNA content of tears was performed from the same group of patients with tear-fluid samples collected from the other eye. Quantitative analysis of RNA between 10–40 nucleotides (nt) in size revealed that tear fluid from AD patients contained significantly higher concentration of microRNA-sized molecules when compared to MCI and control (Fig. 5a,b). The extraction of microRNAs from 200 µl of diluted tear fluid produced on average 7.87 ng of RNA from AD patients, 4.78 ng in samples from patients with MCI and 3.79 ng in control (Fig. 5c). Varying volumes of tear fluid were collected from each of the volunteers ranging from 1–35 mm. The quantity of tear fluid sample showed no significant difference between conditions (p = 0.734) and no correlation with concentrations obtained after RNA extraction to mm of tear fluid obtained by Schirmer strip was observed (r^2^ = 0.010).

These results suggest quantitative alterations of small RNA levels occur within tear fluid of AD patients.

### Profiling of microRNAs in tear fluid of AD patients

**Figure 6:**
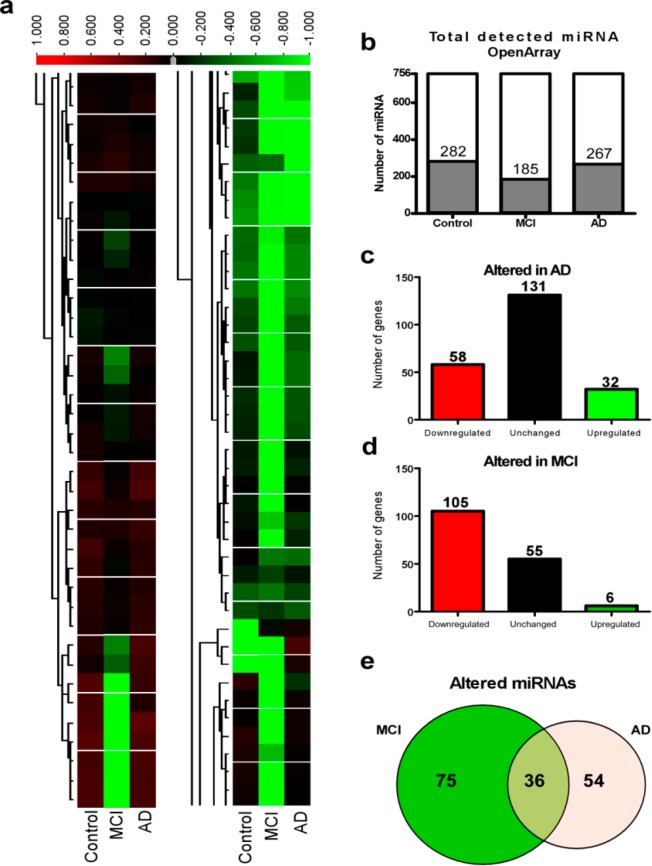
OpenArray-based quantification of microRNA profile in tears of patients with MCI and AD. (**a**) Heatmap of microRNA profile of tear samples from control (Con), and patients with mild cognitive impairment (MCI) and Alzheimer’s disease (AD). (**b**) Total number of microRNAs detected by the OpenArray within each condition. (**c**,**d**) Altered levels of microRNAs in patients suffering from AD (**c**) and MCI (**d**) relative to control. (**d**) Venn Diagram of the total number of altered microRNAs within MCI and AD groups and the overlap of altered microRNAs between the 2 conditions.

To identify distinct microRNAs present in AD, MCI and control patient tear fluid, we used a genome-wide high-throughput qPCR-based microRNA platform (OpenArray) which allows us to examine the expression of 750 microRNAs in humans^34^. This analysis was performed on each condition using a pool of three samples. To avoid a possible bias in our analysis, we chose samples with a consistently high concentration of small RNAs (10–40 nt) (Fig. 6a). Demonstrating the correct functioning of the OpenArray, amplification was observed in all internal positive controls (RNU48, RNU44 and U6) while the negative control ath-miR159a was undetectable.

Using a cut-off of 35 cycle threshold (CT), analysis of the OpenArray detected 282 microRNAs in control, 200 microRNAs in MCI and 266 microRNAs in AD samples (Fig. 6b). Of these, 162 microRNAs were common to all three conditions (Supplementary File 1). Among microRNAs detectable across the 3 conditions, the 5 most abundant microRNAs were microRNA-338-5p, microRNA-378, microRNA-657, microRNA-1243 and microRNA-1247b (Supplementary File 1). In total 194 individual microRNAs were identified with a difference of >1.5 or <0.6 relative to control levels between MCI (156 microRNAs) and AD (102 microRNAs). Interestingly, down-regulation of microRNAs was the main response in both MCI (105 down-regulated microRNAs vs. 6 upregulated microRNAs) and AD (58 down-regulated microRNAs vs. 32 upregulated microRNAs) compared to control (Fig. 6d). Numerous other microRNAs were exclusively detected in AD (38 microRNAs) and/or MCI (14 microRNAs) which included miR-200b-5p which was only detected in AD samples (Supplementary File 1).

### MicroRNA-200b-5p in tear fluid as a potential biomarker of AD

**Figure 7:**
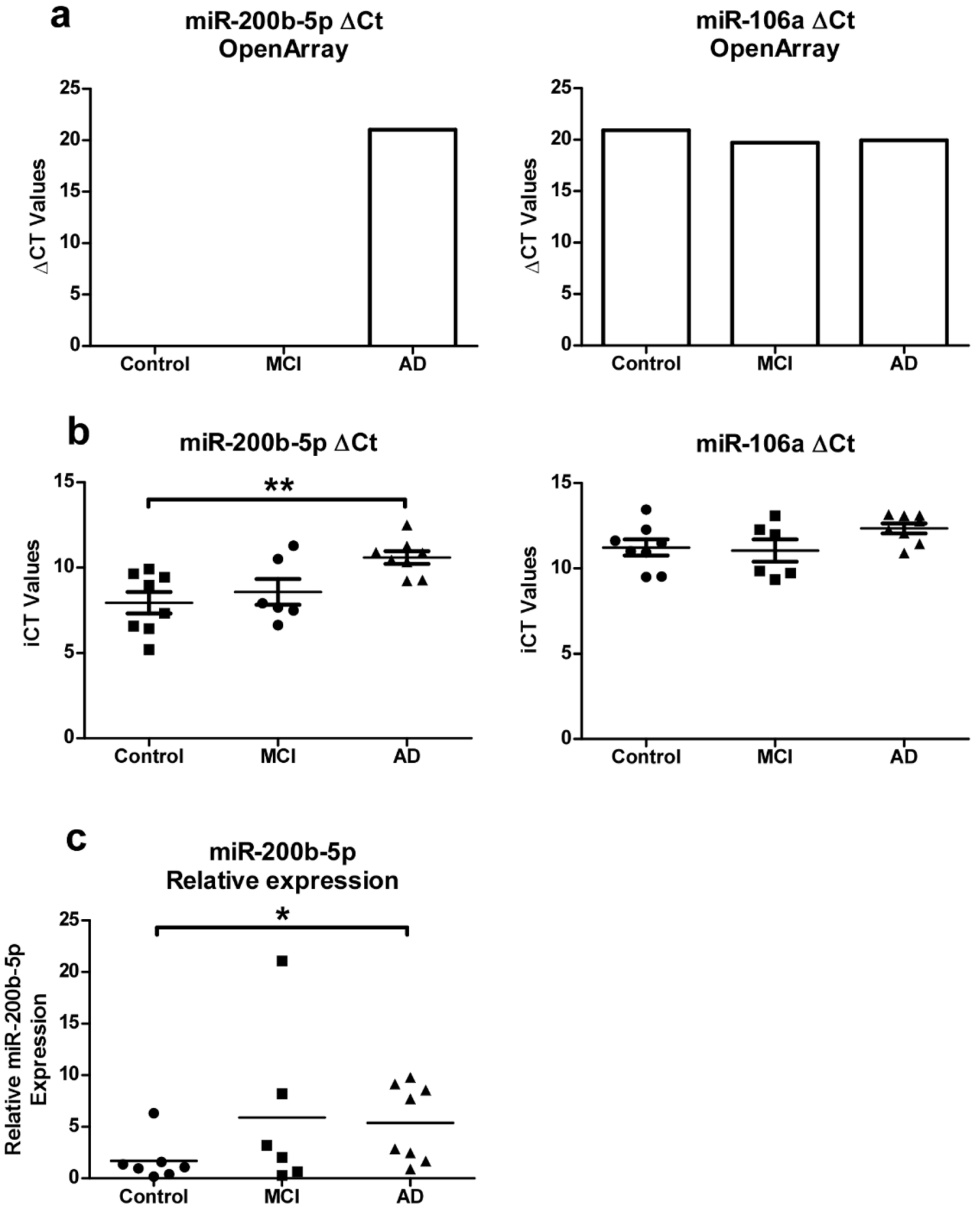
Individual validation of disease-specific microRNA concentration changes within tear fluid. (**a**) Inverted Ct values (iCT = 40 − Ct) of microRNA-200b-5p and reference gene microRNA-106a detected by OpenArray. MicroRNA-200b was undetectable within control samples by the OpenArray. (**b**) Raw iCt values of microRNA-200b-5p and microRNA-106a by individual Taqman RT-qPCR between control (n = 8), MCI (n = 6) and AD (n = 6). (**c**) Relative expression of microRNA-200b-5p normalised to reference gene microRNA-106a (Mann-Whitney; *p < 0.05, **p < 0.01).

MicroRNA-200b-5p was selected from the OpenArray profile for verification as potential biomarker for AD using individual RT-qPCRs. MicroRNA-200b-5p was chosen because it was exclusively detected in AD samples by the OpenArray (Fig. 7a). Notably microRNA-200b-5p was not detected in the only previous microRNA profile of tears which analysed young healthy control subjects, further suggesting that miR-200b-5p expression is distinct in tear fluid of AD patients^31^. To date, microRNA-200b-5p, however, has not been analysed as potential biomarker for AD. Due to the lack of an established control for normalization of microRNAs in tear fluids, we chose microRNA-106a as endogenous control microRNA showing a high expression as detected by the OpenArray with minimal differences between groups (Fig. 7a). Quantification of microRNA-200b-5p by individual RT-qPCR confirmed our OpenArray results with higher levels present in tear fluid of AD patients (Fig. 7b). Further, as expected, microRNA-106a showed similar levels between samples from control, MCI and AD patients demonstrating its suitability as control for normalization (Fig. 7b). Finally, when normalizing microRNA-200b-5p expression to microRNA-106, patients with AD showed a significant increase when compared to control (Fig. 7c).

Taken together, these findings suggest the existence of distinct microRNA changes in tear fluid occurring during AD. This study establishes a strong precedent for further study of microRNAs in tear fluid within AD and other neurological diseases with increased cohorts of patients as potential source of minimally invasive biomarkers.

## Discussion

The present study suggests that a unique proteomic and microRNA composition may be present in tear fluid of AD patients. Using proteomic analysis, we identified 12 proteins contained within the majority of AD samples and only present in a small minority of control samples (Table 1). Analysis of the microRNA composition in our AD group also revealed increases in total concentration of microRNAs within tear fluid as well as distinct changes in the relative expression of common microRNAs including microRNA-200b-5p. The current study establishes, therefore, a strong rationale for further investigation into biomarkers within tear fluid, in particular with the novel finding of altered microRNA content in tear fluid of AD patients.

Previous studies have shown extensive ocular alterations related to AD^35,36^ and that changes in cellular components present in tear-fluid may be used to diagnose AD^18^. Human tear proteins have been separated and identified in the past by using several analytical approaches such as SDS-PAGE and Western blot as well as mass-spectrometry techniques^18^. Neither tau nor β-amyloid peptides have been identified in our study which is notable for β-amyloid due to its invasive nature and widespread presence in various non-brain tissues^37^. Full-length β-APP has, however, been recognized by Western blot using different β-amyloid-specific antibodies in human tear fluid samples previously^38^. MS has been employed previously to study altered protein expression in AD using cortical brain tissue, CSF and even tear fluid^18,39,40^. Notably this previous investigation of tear fluid by MS was performed on excised bands that were separated out by SDS-PAGE and showed significant intensity difference between AD, thus giving a focused MS analysis on a subset of proteins in tears. This strategy is distinct from our study in which MS was performed on the total protein samples of tear fluid, allowing analysis of the total proteomic content of tear fluid in AD.

In our study, in line with previous reports^18^, we have not observed a significant increase in tear flow rate and tear protein concentration, although an increase in the total number of distinct proteins present in tear samples from AD patients suggest that some differences in tear fluid protein composition exist. Notably, a large number of proteins were present only in few samples and, although the source of these sporadically present proteins is unknown, we speculate that slight epithelial damage caused by filter paper-based sampling might have contributed to the contamination of samples along with disintegration of cellular contamination during the freeze-thaw cycle that takes place during transport of the samples as has been previously proposed^41^. The intracellular proteins detected as differentially expressed might be due to the Schirmer paper method itself because this method contains tear fluid and possibly the cells as well. Thus, in addition to the individual variability of tear proteins, this may contribute to the inconsistent protein detection in the samples.

We identified a panel of 12 proteins differentially expressed in tear fluid of AD patients when compared to controls. Among these proteins is eIF4E, which recognizes and binds the 7-methylguanosine-containing mRNA cap during the initiation of protein synthesis facilitating ribosome binding. Previous data has shown an increase of phosphorylated eIF4E in brain tissue of AD patients^42^. Moreover, a reduction of CYFIP1 levels, a protein that represses cap-dependent translation of mRNA by interacting with the initiation factor eIF4E, triggers a cascade of changes towards AD^43^. Interestingly, protein levels of translation initiation factors including eIF2a, eIF3η, eIF5, and elongation factor eIF2, are altered in the hippocampus of AD patients^44^. Complement activation occurs in the brain of patients with AD and contributes to the development of an inflammatory state^45^. In our study we have detected two related proteins; complement factor H-related protein 1^46^ and complement component C8 alpha chain. SERPINB3 seems to be enriched in AD tear fluid samples and this protein and other serpins are found within the fibrillary amyloid plaques of brains from patients with AD^47^. However, exactly how these 12 proteins are mechanistically involved in AD remains unclear. Notably 4 of the 12 proteins are among the 1651 proteins previously differentially expressed in various regions of the brain by MS; Serine-threonine kinase receptor-associated protein, Complement factor H-related protein 1, Tropomyosin alpha-4 chain and Complement component C8 alpha chain^48^. Asparagine-tRNA ligase, cytoplasmic and Corticosteroid-binding globulin were not detected in AD and the remaining 6 were unchanged from controls^48^.

Although this is the first investigation of microRNAs in tear fluid of subjects with a neurological condition, microRNAs as biomarkers have been extensively investigated in blood and CSF. CSF is the optimal biofluid for the study of neurological diseases with it directly interacting with neuronal tissues. Several putative microRNA biomarkers have been identified in CSF but its utility is ultimately limited by the practicality of its invasive method of collection^49^. Blood, on the other hand, only requires a minimally invasive collection which has led to it being the primary focus in investigating microRNA-based biomarkers for AD with some success^50–52^. One major complication to the use of blood-based microRNAs for neurological conditions is its circulating nature with blood interacting with almost every tissue in the body and thus potentially absorbing microRNAs released from these tissues. This is an issue particularly in age-related diseases such as AD where co-morbidities are common further complicating the identification of microRNA changes unique to a neurological disorder^52^. Tears may present a biofluid which avoids both of these complications with non-invasive collection and relatively isolated interactions. Quantitative analysis of microRNAs in tear samples revealed significantly higher concentrations of microRNA-sized molecules present within AD patients compared to control. The reason for the increased presence of microRNAs in AD is unclear but microRNA expression is known to change rapidly in response to cellular disruptions and is likely to be affected by the numerous degenerative processes associated with AD^53–55^. Increased degeneration of the eye through choroidal thinning and retinal degeneration all occur at increased rates within AD with putative links to AD pathology^56,57^. The destructive processes involved in addition to increased neuro-inflammatory response in AD could potentially drive the cellular responses which increase the expression of microRNA found in AD tears^56,57^.

Tear fluid is a seldom investigated biofluid and currently only one previous study has analysed microRNA content in tears^31^. In this study, microRNAs from tear fluid samples of 5 healthy donors were pooled and analysed by miScript microRNA QC PCR Array (Qiagen). A total of 320 microRNAs were detected, compared to the 284 microRNAs within our OpenArray of control samples with 133 microRNAs common between both profiles. The large percentage of miRNAs present in both profiles may indicate a more representative population of microRNAs consistently present in tear fluid.

MicroR200b-5p was verified as a potential novel biomarker for AD found to be elevated in tear fluid of AD patients. Importantly, when quantified by individual RT-qPCR, results mirrored observed changes within the OpenArray. MicroRNA-200b-5p’s function or relationship with AD or any process related to the eye or CNS is relatively unknown with it only having been studied functionally in oncogenic tissue with putative targeting of several elements of Ras homolog gene family pathways. It was also identified as an enriched microRNA within exosomes of aged mice which could contribute to microRNA-200b-5p’s increased presence in tear fluid with the ease at which exosomes pass membrane barriers facilitating its entry into tear fluid^49^. Notably microRNA-200b’s more commonly expressed isomiR miR-200b-3p^58^, was present at high concentrations in both conditions. MiR-200b-3p has putative roles in reducing beta-amyloid toxicity^59^ as well as potential biomarker in serum for AD^52^ but showed no significant difference between conditions in the OpenArray.

Results in total microRNA concentration and microRNA-200b-5p levels showed, however, no significant differences between MCI compared to control and with only limited differences identified in their proteomic composition. This was not entirely surprising with MCI being a heterogeneous condition both occurring as a result of neurodegeneration during the prodromal stage of most neurodegenerative diseases as well as cognitive impairments due to natural aging^3^. Future studies should be undertaken with larger MCI patient cohorts, preferably from longitudinal studies with a confirmed conversion from MCI to AD. Nevertheless, despite the limited sample size of our profiling study, our findings have demonstrated tear fluids as a source of highly concentrated microRNA and identified that both total concentration of microRNA and individual microRNAs within tear fluid become altered in AD. Thus, the development of novel qPCR-independent microRNA-measuring devices, already in development^60^ may be a plausible new approach for a quick and non-invasive detection of AD development.

In conclusion, we have demonstrated that tears, collected with a minimal discomfort for the patient, are an appropriate source of microRNA and may be used along with tear proteins as biomarkers for AD. The identification of these biomarkers in tear samples collected with Schirmer strips may allow for the development of a simple, non-invasive and cost-effective test for AD. Future studies with a greater sample size should be completed to replicate and extend these findings.

## Methods

### Sample collection

The present study consisted of 32 donors (11 men [36.4%] and 21 women) including 9 patients with AD, 8 with MCI and 15 age-matched controls. Table 2 describes the demographics of participants. All age-matched controls (relatives of the patients) were seen by a neurologist, who did an interview and a complete physical examination ruling out any clinical neurological disease. Sample collection complied with the guidelines of the Helsinki Declaration and ethical approval was obtained from the Carlos III Institute of Health Ethics Committee (No. CEI PI 20_2014), while subjects gave informed written consent. In the case of AD patients, the consent was acquired in the presence of a caregiver, although none of them were placed under guardianship. All donors were from Soria (Spain) and the assessment of AD was carried out by a neurologist. MCI was based on Petersen criteria^61^ and AD was based on the NIA-AA criteria^62^. The diagnosis was AD with no biomarkers (imaging or biofluids) available. Besides neurological, neuropsychological and functional assessment, patients underwent an ophthalmic revision.

**Table 2:** Human samples used in the study.

Sample and birth year	Gender	Time (min)	Tear flow (mm)	Ophthalmic allergy	Ophthalmic infection	Ocular hypertension	Refractive defect	Dry eye	cataracts	
02 S-1933	M	5	3	0	0	0	0	0	1	MCI
03 S-1937	F	5	20	0	0	0	1	0	1	CONTROL
04 S-1926	F	5	25	0	0	0	0	0	0	CONTROL
05 S-1929	F	5	9	0	0	0	1	0	0	ALZHEIMER
06 S-1925	M	2,2	35	0	0	1	0	0	1	CONTROL
07 S-1934	F	5	8	0	0	0	1	0	1	MCI
08 S-1938	F	5	2	0	0	0	1	1	1	CONTROL
09 S-1934	M	5	18	0	0	1	1	0	1	MCI
10 S-1936	F	5	5							ALZHEIMER
11 S-1929	F	5	6							ALZHEIMER
12 S-1925	M	5	11	0	0	0	1	0	0	CONTROL
13 S-1932	F	5	13	0	0	0	1	0	1	MCI
14 S-1938	F	5	7	0	0	0	1	0	0	ALZHEIMER
15 S -1928	F	5	2	0	1	0	0	0	1	CONTROL
16 S-1925	F	5	13	1	1	1	1	0	1	CONTROL
17 S-1933	F	5	7	0	0	0	1	0	1	CONTROL
18 S-1935	M	5	8	0	0	0	0	0	1	ALZHEIMER
19 S-1941	F	5	2	1	0	0	0	0	1	CONTROL
20 S-1940	F	5	14	0	0	0	1	0	0	CONTROL
21 S-1935	M	5	14	0	0	0	0	0	0	ALZHEIMER
22 S-1933	M	5	5	0	0	1	0	0	1	CONTROL
24 S-1944	M	5	11	0	0	0	0	0	0	ALZHEIMER
25 S-1940	F	5	11	0	0	0	1	0	1	CONTROL
26 S-1943	F	5	3	1	0	0	1	0	0	CONTROL
27 S-1939	M	5	10	0	0	1	0	0	1	CONTROL
28 S -1932	F	5	18	0	0	0	1	0	1	MCI
29 S -1928	F	5	8							ALZHEIMER
30 S-1940	M	5	4	0	0	0	1	0	0	MCI
31 S-1936	F	5	19							CONTROL
32 S-1926	F	5	3	0	0	0	0	0	1	MCI
33 S-1940	F	5	7							MCI
34 S-1929	M	5	1							ALZHEIMER

Tear secretion was measured by using the Schirmer’s Test (Schirmer strips; Whatman, Maidstone, UK). Schirmer’s paper was kept in the lower lid margin for 5 min. Tear secretion was measured as the length of the wet strip (in mm). The right eye was used to analyse protein content while the left eye was used to analyse microRNA content. Schirmer’s tear fluid collection was carried out simultaneously in both eyes. Paper strips from the right eyes were then placed in Eppendorf tubes containing 500 μl of PBS with a cocktail of peptidase inhibitors (Roche, Basel Switzerland), strongly vortexed for 5 min and frozen in dry ice. Protein content was determined by the Bradford method (Bio-Rad, Hercules, CA, USA). The paper strips from left eyes were also placed in Eppendorf tubes containing 500 μl of water with RNase inhibitors (Diethyl pyrocarbonate (DEPC)-treated water, Ambion, CA, United States) and strongly vortexed for 5 min and frozen in dry ice. Upon arrival to the laboratory both samples were stored at −80 °C.

### Protein analysis by RP-LC-MS/MS

In-Gel Digestion (Stacking gel): 100 µl of tear samples were mixed with sample buffer (4x) and boiled for 2 min. 50 µl of samples were then applied onto 1.2-cm wide wells of a conventional SDS-PAGE gel (0.75 mm-thick, 4% stacking, and 10% resolving). The run was stopped as soon as the front entered 3 mm into the resolving gel, so that the whole proteome became concentrated in the stacking/resolving gel interface. The unseparated protein bands were visualized by Coomassie staining, excised, cut into cubes (2 × 2 mm), and placed in 0.5 ml microcentrifuge tubes^63^. The gel pieces were destained in acetonitrile:water (ACN:H2O, 1:1), reduced and alkylated (disulfide bonds from cysteinyl residues were reduced with 10 mM DTT for 1 h at 56 °C, and thiol groups were alkylated with 50 mM iodoacetamide for 1 h at room temperature in darkness) and digested *in situ* with sequencing grade trypsin (Promega, Madison, WI) as described by Shevchenko *et al*.^64^ with minor modifications. The gel pieces were shrunk by removing all liquid using sufficient ACN. Acetonitrile was pipetted out and gel pieces were dried in a speedvac. The dried gel pieces were re-swollen in 50 mM ammonium bicarbonate pH 8.8 with 60 ng/µl trypsin at 5:1 protein:trypsin (w/w) ratio. The tubes were kept in ice for 2 h and incubated at 37 °C for 12 h. Digestion was stopped by the addition of 1% TFA. Whole supernatants were dried down and then desalted onto OMIX Pipette tips C18 (Agilent Technologies) before the mass spectrometric analysis was carried out.

Reverse phase-liquid chromatography RP-LC-MS/MS analysis (Dynamic Exclusion Mode): The desalted protein digest was dried, suspended in 10 µl of 0.1% formic acid and analyzed by RP-LC-MS/MS in an Easy-nLC II system coupled to an ion trap LTQ-Orbitrap-Velos-Pro hybrid mass spectrometer (Thermo Scientific). The peptides were concentrated (on-line) by reverse phase chromatography using a 0.1 mm × 20 mm C18 RP precolumn (Thermo Scientific), and then separated using a 0.075 mm × 250 mm C18 RP column (Thermo Scientific) operating at 0.3 μl/min. Peptides were eluted using a 180-min dual gradient from 5 to 25% solvent B in 135 min followed by gradient from 25 to 40% solvent B over 180 min (Solvent A: 0,1% formic acid in water, solvent B: 0,1% formic acid, 80% acetonitrile in water). ESI ionization was carried out using a Nano-bore emitters Stainless Steel ID 30 μm (Proxeon) interface. The Orbitrap resolution was set at 30.000. Peptides were detected in survey scans from 400 to 1600 amu (1 μscan), followed by twenty data dependent MS/MS scans (Top 20), using an isolation width of 2 u (in mass-to-charge ratio units), normalized collision energy of 35%, and dynamic exclusion applied during 30 seconds periods. Peptide identification from raw data was carried out using the SEQUEST algorithm (Proteome Discoverer 1.4, Thermo Scientific) and PEAKs 8.5 software. Database search was performed against Uniprot_Homo_sapiens_SwissProt. The following constraints were used for the searches: tryptic cleavage after Arg and Lys, up to two missed cleavage sites, and tolerances of 20 ppm for precursor ions and 0.8 Da for MS/MS fragment ions and the searches were performed allowing optional Met oxidation, Cys carbamidomethylation and Ser-Thr-Tyr Phosphorylation. Search against decoy database (integrated decoy approach) was carried out using false discovery rate (FDR) < 0.01. The list of identified proteins with at least two identified peptides are provided as Supplementary Data (Supplementary File 2). The mass spectrometry proteomics data have been deposited to the ProteomeXchange Consortium via the PRIDE partner repository with the dataset identifier PXD014475^65^.

### MicroRNA extraction

Small RNAs were isolated from patient tear fluid using a miRNeasy Serum/Plasma kit following manufacturer’s instruction. Briefly, RNA were isolated by Qiazol/chloroform phase separation, then small RNA were further isolated by serum/plasma small RNA kit (Qiagen, West Sussex, UK) and diluted in 142 μl of RNase free water. 2 µl of purified small RNA isolate was analysed on AATI Fragment analyser (AATI, Iowa USA) for quantification of small RNA concentration. Quantity of RNA was assessed on the AATI Fragment analyser considering the range of 10–40 nucleotide long molecules as miRNAs. For profiling on the OpenArray, the three samples with higher concentration of small RNA in each condition were selected and pooled by condition.

### OpenArray microRNA profiling

MicroRNA profiling was performed using the OpenArray platform from Thermo Fisher, as described previously^66^. Briefly, the OpenArray reverse transcription reaction was performed according to the manufacturer’s protocol using 3 µl of total RNA pooled from 3 samples. Before loading onto the OpenArray, cDNA was pre-amplified following the manufacturer’s recommendation. Then, the pre-amplified product was diluted with 0.1X TE (1/40) and 22.5 µl of diluted pre-amplified product was added to the same volume of 2X Taqman OpenArray Real time PCR Master Mix (Cat No. 4462164, AB). Finally, the mix of Pre-Amp product and Master-Mix was loaded onto a 384-well OpenArray plate. OpenArray panels were automatically loaded by the OpenArray AccuFill System (Thermo Fisher Scientific, Waltham, Massachusetts) and run on a QuantStudio 12 K Flex Real-Time PCR system. Each panel enables the quantification of microRNA expression of three samples. 754 microRNAs were amplified from each sample together with 16 replicates of four internal controls (ath-miR159a (negative control), RNU48, RNU44 and U6 rRNA).

### Analysis of microRNA profile

The resulting data from OpenArray profiling was processed by an initial cleaning step with data being filtered according to “AmpScore” or “CqConf” values provided by Expression Suite software (Thermo Fisher Scientific, Waltham, Massachusetts) to avoid false positives. Cut off thresholds of 35 Ct were considered to be absent. After the initial cleaning, the OpenArray qPCR profile were normalised based on Geometric Mean Normalization, one of the most reliable normalisation strategies available and was performed as previously described^67^ with the normalised Ct (ΔCt) calculated as ΔCt = Ct_microRNA_ − Ct_Global-Mean_. The normalised MCI and AD patient samples were then quantified as relative expression to the mean of the normalised control. Verification of OpenArray was performed within a separate cohort of patients and quantified by Taqman small-scale microRNA assays^34^ on 1.69 µl of each sample. Each of the selected microRNAs were reverse transcribed by Taqman microRNA reverse transcription kit with Taqman assay primers specific and quantified with qPCR Taqman fast universal master mix qPCR in a Quant Studio 12 K Flex real-time qPCR system. QPCR was performed in triplicate with a negative control for each qPCR. This protocol was followed to quantify microRNA levels of microRNA-200b-5p (Thermofischer scientific, assay ID: 478753). All Ct values were normalised to miR-106a (Thermofischer scientific, assay ID: 478225) by ΔCt method. Relative expression (RE) was calculated as RE = 2^−ΔΔCt^, where ΔΔCt = ΔCt_target microRNA_ − ΔCt_reference gene_.

### Statistical analysis

Comparison of groups was performed using ANOVA test and post unpaired t-test. Kurskal-wallis followed by Dunn’s post-hoc test was carried out to test for significance between groups for a specific microRNA. Significance was accepted at p < 0.05.

## Supplementary information

Supplementary Figure 1

Supplementary file 1

Supplementary file 2
