# Supplementary material for: Proteins and microRNAs are differentially expressed in tear fluid from patients with Alzheimer’s disease
Source: Sci Rep. 2019 Oct 28;9:15437. doi: 10.1038/s41598-019-51837-y (PMC6817868; doi:10.1038/s41598-019-51837-y)
Supplement: Supplementary file 1 — Supplementary Figure 1 [file 41598_2019_51837_MOESM1_ESM.pdf]

Supplementary Figure 1

Proteins and microRNAs are differentially expressed in tear fluid from patients with Alzheimer's disease

Aidan Kenny, Eva M. Jiménez-Mateos, María Ascensión-Zea, Alberto Rábano, Pablo Gili-Manzanaro, Jochen H.M. Prehn, David C. Henshall, Jesús Ávila, Tobias Engel, Félix Hernández

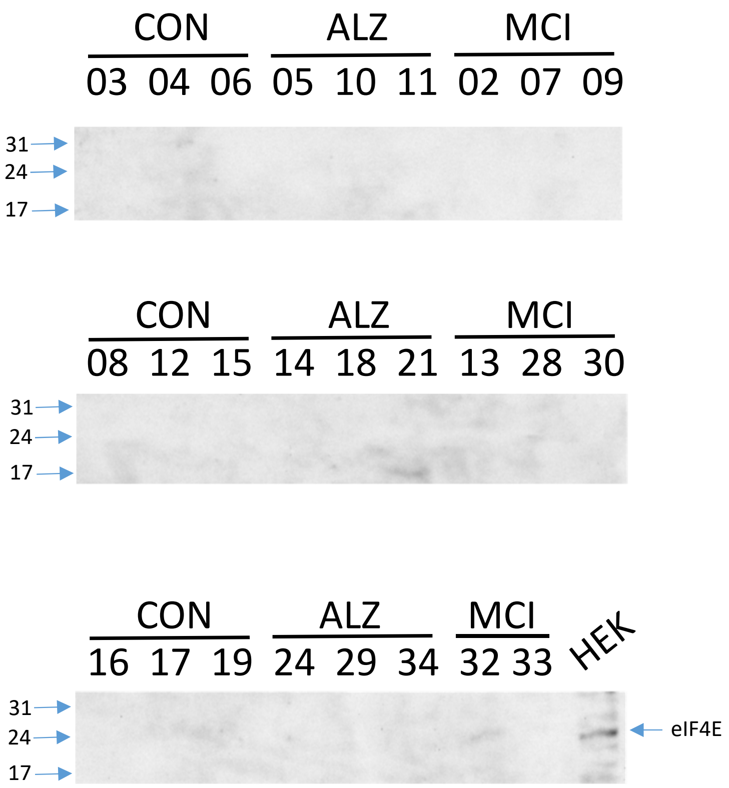

Supplementary Fig. 1.

Tear proteins (3 µg per sample) were electrophoresed on 12% SDS-polyacrylamide gel and transferred to a nitrocellulose membrane (Schleicher & Schuell). Prior to antibody hybridation, membranes were blocked with 5% nonfat dried milk. The primary antibody used was rabbit anti-EIF4E (1:500 SIGMA; catalog no. E5906, lot 066M4877V). The membranes were incubated with the antibody at 4 °C overnight in the same blocking solution. Secondary goat anti-rabbit antibody (1:5000; Dako) conjugates with HRP and ECL detection reagents (Amersham Biosciences) were used for immunodetection. Numbers of CON, ALZ and MCI samples correspond to those samples shown in Table II. Lysates from HEK-293 cells were used as positive control.
