# Supplementary material for: Proteins and microRNAs are differentially expressed in tear fluid from patients with Alzheimer’s disease
Source: Sci Rep. 2019 Oct 28;9:15437. doi: 10.1038/s41598-019-51837-y (PMC6817868; doi:10.1038/s41598-019-51837-y)
Supplement: Supplementary file 2 — Supplementary file 1 [file 41598_2019_51837_MOESM2_ESM.docx]

Supplementary Information

**Proteins and microRNAs are differentially expressed in tear fluid from patients with Alzheimer’s disease**

Aidan Kenny, Eva M. Jiménez-Mateos, María Ascensión-Zea, Alberto Rábano, Pablo Gili-Manzanaro, Jochen H.M. Prehn, David C. Henshall, Jesús Ávila, Tobias Engel, Félix Hernández

*Supplementary file 1* : Raw Ct values of detected microRNAs for each condition on the Open Array platform. Blue: MicroRNAs expressed in all 3 conditions. Orange: microRNAs present in 2 out of 3 conditions. Yellow: microRNAs only detected in one conditions. Control: Control. MCI: Mild Cognitive Impairment. AD: Alzheimer’s Disease

| **miRNA ID** | **Control** | **MCI** | **AD** |
| --- | --- | --- | --- |
| **000379_hsa-let-7c_A** | 22.249 | 24.819 | 22.727 |
| **000391_hsa-miR-16_A** | 17.761 | 21.208 | 19.506 |
| **000395_hsa-miR-19a_A** | 25.615 | 34.672 | 26.688 |
| **000396_hsa-miR-19b_A** | 18.685 | 21.921 | 20.100 |
| **000397_hsa-miR-21_A** | 19.222 | 23.088 | 20.996 |
| **000402_hsa-miR-24_A** | 14.189 | 16.327 | 14.693 |
| **000405_hsa-miR-26a_A** | 20.022 | 24.565 | 21.802 |
| **000407_hsa-miR-26b_A** | 21.758 | 26.796 | 22.588 |
| **000408_hsa-miR-27a_A** | 23.651 | 27.449 | 25.181 |
| **000411_hsa-miR-28_A** | 22.775 | 26.169 | 23.806 |
| **000413_hsa-miR-29b_A** | 26.144 | 27.222 | 26.464 |
| **000416_hsa-miR-30a-3p_B** | 20.545 | 24.756 | 20.526 |
| **000417_hsa-miR-30a-5p_B** | 18.538 | 23.373 | 17.683 |
| **000419_hsa-miR-30c_A** | 17.832 | 21.513 | 18.980 |
| **000420_hsa-miR-30d_B** | 21.128 | 26.760 | 20.007 |
| **000422_hsa-miR-30e-3p_B** | 19.798 | 21.918 | 19.982 |
| **000431_hsa-miR-92a_A** | 21.632 | 27.333 | 23.783 |
| **000433_hsa-miR-95_A** | 21.985 | 24.835 | 22.148 |
| **000435_hsa-miR-99a_A** | 22.013 | 23.625 | 23.785 |
| **000436_hsa-miR-99b_A** | 20.987 | 24.684 | 21.918 |
| **000437_hsa-miR-100_A** | 22.193 | 25.619 | 23.984 |
| **000439_hsa-miR-103_A** | 21.777 | 25.258 | 23.363 |
| **000442_hsa-miR-106b_A** | 21.692 | 25.793 | 22.767 |
| **000449_hsa-miR-125b_A** | 21.177 | 24.476 | 21.535 |
| **000460_hsa-miR-135a_A** | 27.832 | 30.138 | 30.252 |
| **000463_hsa-miR-141_A** | 18.730 | 21.825 | 19.223 |
| **000464_hsa-miR-142-3p_A** | 20.823 | 28.212 | 25.059 |
| **000468_hsa-miR-146a_A** | 12.531 | 15.319 | 13.126 |
| **000470_hsa-miR-148a_A** | 24.690 | 26.943 | 25.332 |
| **000473_hsa-miR-150_A** | 14.993 | 19.148 | 17.449 |
| **000475_hsa-miR-152_A** | 22.724 | 25.727 | 23.156 |
| **000480_hsa-miR-181a_A** | 24.600 | 29.926 | 25.828 |
| **000483_hsa-miR-182#_B** | 28.693 | 30.898 | 27.060 |
| **000485_hsa-miR-184_A** | 12.223 | 13.415 | 12.776 |
| **000491_hsa-miR-192_A** | 22.080 | 26.500 | 23.536 |
| **000493_hsa-miR-194_A** | 25.314 | 23.552 | 26.641 |
| **000494_hsa-miR-195_A** | 24.222 | 27.618 | 25.740 |
| **000497_hsa-miR-197_A** | 18.716 | 21.604 | 20.131 |
| **000502_hsa-miR-200a_A** | 17.366 | 19.843 | 17.817 |
| **000507_hsa-miR-203_A** | 14.687 | 17.644 | 15.396 |
| **000508_hsa-miR-204_A** | 20.269 | 21.211 | 19.269 |
| **000512_hsa-miR-210_A** | 19.508 | 21.842 | 20.194 |
| **000514_hsa-miR-211_A** | 20.594 | 21.658 | 19.751 |
| **000515_hsa-miR-212_A** | 23.406 | 25.343 | 23.764 |
| **000518_hsa-miR-215_A** | 22.009 | 25.451 | 24.417 |
| **000521_hsa-miR-218_A** | 23.820 | 28.869 | 24.525 |
| **000543_hsa-miR-328_A** | 25.137 | 26.655 | 25.369 |
| **000544_hsa-miR-330_A** | 25.649 | 28.544 | 26.749 |
| **000545_hsa-miR-331_A** | 17.592 | 20.090 | 18.265 |
| **000563_hsa-miR-374_A** | 20.739 | 25.661 | 21.555 |
| **000564_hsa-miR-375_A** | 19.750 | 21.784 | 22.027 |
| **000587_hsa-miR-29c_A** | 17.785 | 19.718 | 17.158 |
| **001006_RNU48_B** | 15.444 | 16.684 | 16.605 |
| **001006_RNU48_B** | 15.449 | 17.148 | 16.620 |
| **001006_RNU48_B** | 15.482 | 17.098 | 16.716 |
| **001006_RNU48_B** | 15.515 | 16.483 | 16.749 |
| **001006_RNU48_B** | 15.615 | 17.336 | 16.724 |
| **001006_RNU48_B** | 15.679 | 17.405 | 16.816 |
| **001006_RNU48_B** | 15.710 | 16.825 | 16.826 |
| **001006_RNU48_B** | 15.713 | 16.787 | 16.796 |
| **001006_RNU48_B** | 15.752 | 17.292 | 16.722 |
| **001006_RNU48_B** | 15.782 | 17.431 | 16.765 |
| **001006_RNU48_B** | 15.783 | 17.338 | 16.837 |
| **001006_RNU48_B** | 15.792 | 17.647 | 17.087 |
| **001006_RNU48_B** | 15.797 | 16.806 | 16.869 |
| **001006_RNU48_B** | 15.798 | 17.616 | 16.952 |
| **001006_RNU48_B** | 15.807 | 16.791 | 16.875 |
| **001006_RNU48_B** | 15.808 | 16.723 | 16.755 |
| **001011_hsa-miR-200a#_B** | 22.603 | 24.125 | 23.110 |
| **001014_hsa-miR-20b_A** | 24.059 | 28.221 | 25.697 |
| **001020_hsa-miR-365_A** | 25.396 | 33.804 | 27.034 |
| **001024_hsa-miR-429_A** | 23.763 | 28.377 | 26.140 |
| **001039_hsa-miR-492_A** | 26.689 | 27.624 | 26.847 |
| **001090_mmu-miR-93_A** | 23.420 | 26.002 | 24.228 |
| **001094_RNU44_B** | 20.905 | 24.796 | 21.321 |
| **001094_RNU44_B** | 21.147 | 24.348 | 21.394 |
| **001094_RNU44_B** | 21.155 | 25.565 | 20.829 |
| **001094_RNU44_B** | 21.169 | 26.680 | 21.753 |
| **001094_RNU44_B** | 21.172 | 25.920 | 21.514 |
| **001094_RNU44_B** | 21.257 | 25.965 | 21.742 |
| **001094_RNU44_B** | 21.449 | 25.544 | 21.752 |
| **001094_RNU44_B** | 21.553 | 25.529 | 21.704 |
| **001094_RNU44_B** | 21.558 | 22.577 | 23.647 |
| **001094_RNU44_B** | 21.639 | 23.644 | 23.370 |
| **001094_RNU44_B** | 21.697 | 24.098 | 23.299 |
| **001094_RNU44_B** | 21.787 | 23.342 | 23.614 |
| **001094_RNU44_B** | 21.796 | 23.211 | 23.651 |
| **001094_RNU44_B** | 21.875 | 23.768 | 23.501 |
| **001094_RNU44_B** | 22.490 | 24.408 | 23.724 |
| **001094_RNU44_B** | 26.706 | 23.530 | 23.500 |
| **001097_hsa-miR-146b_A** | 17.419 | 18.601 | 17.867 |
| **001159_hsa-miR-518d_A** | 23.819 | 23.117 | 23.182 |
| **001273_hsa-miR-362_A** | 26.976 | 27.853 | 28.116 |
| **001319_mmu-miR-374-5p_A** | 20.763 | 22.666 | 21.533 |
| **001338_rno-miR-7#_B** | 19.520 | 21.840 | 18.931 |
| **001512_hsa-miR-657_B** | 11.187 | 12.385 | 12.018 |
| **001531_hsa-miR-564_B** | 10.572 | 27.147 | 22.290 |
| **001538_hsa-miR-548a_A** | 26.656 | 26.279 | 26.546 |
| **001553_hsa-miR-622_B** | 25.364 | 27.481 | 24.995 |
| **001558_hsa-miR-601_B** | 28.905 | 30.142 | 27.818 |
| **001562_hsa-miR-629_B** | 22.169 | 24.210 | 23.733 |
| **001566_hsa-miR-603_B** | 27.892 | 27.987 | 29.239 |
| **001590_hsa-miR-548c_A** | 28.146 | 27.300 | 26.939 |
| **001592_hsa-miR-642_A** | 25.397 | 27.849 | 26.311 |
| **001594_hsa-miR-643_B** | 14.508 | 15.262 | 14.818 |
| **001603_hsa-miR-650_B** | 23.835 | 25.625 | 24.094 |
| **001630_mmu-miR-491_A** | 23.804 | 23.979 | 23.991 |
| **001818_rno-miR-29c#_B** | 25.930 | 29.579 | 25.597 |
| **001821_hsa-miR-484_A** | 15.463 | 17.304 | 15.985 |
| **001973_U6 rRNA_B** | 8.147 | 10.418 | 9.507 |
| **001973_U6 rRNA_B** | 8.156 | 10.834 | 9.343 |
| **001973_U6 rRNA_B** | 8.292 | 10.787 | 9.583 |
| **001973_U6 rRNA_B** | 8.341 | 10.668 | 9.760 |
| **001973_U6 rRNA_B** | 8.395 | 10.670 | 9.650 |
| **001973_U6 rRNA_B** | 8.432 | 10.715 | 9.706 |
| **001973_U6 rRNA_B** | 8.442 | 10.707 | 9.691 |
| **001973_U6 rRNA_B** | 8.501 | 10.695 | 9.769 |
| **001973_U6 rRNA_B** | 8.764 | 10.290 | 10.125 |
| **001973_U6 rRNA_B** | 8.802 | 10.744 | 9.884 |
| **001973_U6 rRNA_B** | 8.959 | 10.628 | 10.257 |
| **001973_U6 rRNA_B** | 9.019 | 10.718 | 10.148 |
| **001973_U6 rRNA_B** | 9.152 | 10.773 | 10.060 |
| **001973_U6 rRNA_B** | 9.160 | 10.721 | 10.176 |
| **001973_U6 rRNA_B** | 9.208 | 10.666 | 10.265 |
| **001973_U6 rRNA_B** | 9.410 | 11.180 | 10.263 |
| **001986_hsa-miR-766_B** | 22.803 | 26.644 | 26.141 |
| **001988_hsa-miR-598_A** | 26.093 | 29.306 | 28.709 |
| **002088_hsa-miR-636_A** | 25.581 | 24.383 | 20.700 |
| **002097_hsa-miR-222#_B** | 22.533 | 23.824 | 22.328 |
| **002098_hsa-miR-223#_B** | 24.806 | 28.617 | 26.403 |
| **002102_hsa-miR-34b_B** | 24.992 | 27.667 | 25.287 |
| **002112_hsa-miR-29a_A** | 18.613 | 21.742 | 19.033 |
| **002113_hsa-miR-31#_B** | 22.037 | 26.409 | 20.621 |
| **002139_hsa-miR-93#_B** | 24.312 | 27.259 | 25.220 |
| **002161_hsa-miR-324-3p_A** | 23.510 | 25.095 | 23.148 |
| **002169_hsa-miR-106a_A** | 19.073 | 20.278 | 20.045 |
| **002174_hsa-miR-27b#_B** | 22.639 | 25.805 | 22.627 |
| **002184_hsa-miR-339-3p_A** | 21.778 | 23.169 | 22.781 |
| **002186_hsa-miR-345_A** | 21.855 | 25.309 | 23.475 |
| **002187_hsa-miR-942_B** | 22.377 | 25.782 | 23.724 |
| **002189_hsa-miR-944_B** | 30.970 | 28.899 | 30.379 |
| **002193_hsa-miR-886-5p_A** | 20.076 | 24.407 | 19.818 |
| **002194_hsa-miR-886-3p_A** | 18.838 | 22.474 | 19.645 |
| **002196_hsa-miR-99b#_B** | 19.821 | 20.821 | 20.526 |
| **002198_hsa-miR-125a-5p_A** | 17.558 | 20.517 | 18.133 |
| **002199_hsa-miR-125a-3p_A** | 31.629 | 31.616 | 31.409 |
| **002228_hsa-miR-126_A** | 22.255 | 25.670 | 22.585 |
| **002234_hsa-miR-140-3p_A** | 23.918 | 26.141 | 24.579 |
| **002235_hsa-miR-509-5p_A** | 25.828 | 26.899 | 25.653 |
| **002243_hsa-miR-378_B** | 11.255 | 10.630 | 28.384 |
| **002246_hsa-miR-133a_A** | 25.891 | 26.774 | 26.354 |
| **002250_hsa-miR-193a-3p_A** | 31.109 | 33.785 | 34.912 |
| **002251_hsa-miR-200b_A** | 16.747 | 17.505 | 16.508 |
| **002255_hsa-miR-149_A** | 21.470 | 23.755 | 22.799 |
| **002260_hsa-miR-342-3p_A** | 16.516 | 17.832 | 17.444 |
| **002261_hsa-miR-135b_A** | 20.557 | 22.308 | 20.929 |
| **002269_hsa-miR-183_A** | 26.828 | 27.037 | 27.415 |
| **002270_hsa-miR-183#_B** | 22.333 | 23.809 | 22.895 |
| **002276_hsa-miR-222_A** | 13.118 | 15.809 | 14.511 |
| **002277_hsa-miR-320_A** | 16.760 | 19.086 | 17.341 |
| **002279_hsa-miR-31_A** | 17.780 | 19.773 | 18.371 |
| **002282_hsa-let-7g_A** | 21.007 | 23.656 | 21.912 |
| **002283_hsa-let-7d_A** | 22.743 | 25.173 | 22.800 |
| **002285_hsa-miR-186_A** | 19.334 | 22.525 | 20.156 |
| **002295_hsa-miR-223_A** | 14.729 | 21.624 | 19.140 |
| **002299_hsa-miR-191_A** | 12.391 | 13.319 | 13.357 |
| **002300_hsa-miR-200c_A** | 12.514 | 13.933 | 12.689 |
| **002302_hsa-miR-425#_B** | 25.297 | 26.465 | 26.249 |
| **002305_hsa-miR-30d#_B** | 26.751 | 29.232 | 25.666 |
| **002308_hsa-miR-17_A** | 19.431 | 22.401 | 20.064 |
| **002316_hsa-miR-34a#_B** | 21.492 | 24.676 | 21.460 |
| **002317_hsa-miR-181a-2#_B** | 26.837 | 28.100 | 25.733 |
| **002323_hsa-miR-454_A** | 17.873 | 20.795 | 19.175 |
| **002324_hsa-miR-744_A** | 23.719 | 25.484 | 24.217 |
| **002334_hsa-miR-182_A** | 25.750 | 28.902 | 27.578 |
| **002338_hsa-miR-483-5p_A** | 20.586 | 19.534 | 22.794 |
| **002340_hsa-miR-423-5p_A** | 25.212 | 26.517 | 25.827 |
| **002349_hsa-miR-574-3p_A** | 15.526 | 17.959 | 16.619 |
| **002355_hsa-miR-532-3p_A** | 22.812 | 25.885 | 23.620 |
| **002358_hsa-miR-489_A** | 21.815 | 22.496 | 21.812 |
| **002366_hsa-miR-193b#_B** | 24.100 | 22.223 | 24.160 |
| **002367_hsa-miR-193b_A** | 15.628 | 17.162 | 16.540 |
| **002386_hsa-miR-523_A** | 17.025 | 13.382 | 12.179 |
| **002388_hsa-miR-518f_A** | 13.825 | 15.828 | 11.701 |
| **002393_hsa-miR-520d-5p_A** | 27.891 | 29.708 | 27.211 |
| **002400_hsa-miR-520c-3p_B** | 15.117 | 14.874 | 15.138 |
| **002406_hsa-let-7e_A** | 17.557 | 20.108 | 18.585 |
| **002432_hsa-miR-625#_B** | 22.134 | 24.920 | 22.979 |
| **002433_hsa-miR-628-5p_A** | 21.678 | 21.513 | 21.782 |
| **002434_hsa-miR-628-3p_B** | 22.891 | 25.565 | 24.310 |
| **002438_hsa-miR-21#_B** | 15.570 | 15.914 | 22.215 |
| **002442_hsa-miR-25#_B** | 24.559 | 25.659 | 22.576 |
| **002443_hsa-miR-26a-1#_B** | 25.860 | 27.333 | 28.133 |
| **002445_hsa-miR-27a#_B** | 21.327 | 23.747 | 20.836 |
| **002446_hsa-miR-28-3p_A** | 19.454 | 22.799 | 20.132 |
| **002619_hsa-let-7b_A** | 17.001 | 16.754 | 15.484 |
| **002623_hsa-miR-155_A** | 16.689 | 21.297 | 20.757 |
| **002642_HSA-MIR-151-5P_B** | 26.366 | 30.475 | 27.449 |
| **002743_HSA-MIR-520D-3P_B** | 11.906 | 17.461 | 13.835 |
| **002758_HSA-MIR-1226#_B** | 20.795 | 22.038 | 23.688 |
| **002769_HSA-MIR-1227_B** | 25.107 | 27.137 | 26.633 |
| **002844_HSA-MIR-320B_B** | 25.861 | 28.887 | 26.893 |
| **002854_HSA-MIR-1243_B** | 4.422 | 9.111 | 4.651 |
| **002857_HSA-MIR-663B_B** | 14.333 | 14.090 | 10.093 |
| **002863_HSA-MIR-1290_B** | 24.755 | 26.050 | 25.811 |
| **002883_HSA-MIR-1274A_B** | 16.220 | 19.836 | 13.615 |
| **002884_HSA-MIR-1274B_B** | 10.607 | 13.719 | 7.803 |
| **002895_HSA-MIR-720_B** | 12.423 | 13.449 | 9.123 |
| **002896_HSA-MIR-1260_B** | 18.425 | 20.872 | 16.559 |
| **002897_HSA-MIR-664_B** | 18.532 | 18.841 | 19.100 |
| **002902_HSA-MIR-1300_B** | 27.135 | 31.525 | 26.494 |
| **000377_hsa-let-7a_A** | 20.252 | Undetermined | 21.218 |
| **000390_hsa-miR-15b_A** | 22.355 | Undetermined | 23.919 |
| **000403_hsa-miR-25_A** | 24.737 | Undetermined | 25.313 |
| **000409_hsa-miR-27b_A** | 25.036 | Undetermined | 25.872 |
| **000426_hsa-miR-34a_A** | 20.745 | Undetermined | 16.580 |
| **000454_hsa-miR-130a_A** | 26.867 | Undetermined | 28.268 |
| **000457_hsa-miR-132_A** | 23.070 | Undetermined | 23.315 |
| **000482_hsa-miR-181c_A** | 29.864 | Undetermined | 31.845 |
| **000509_hsa-miR-205_A** | 22.765 | Undetermined | 24.792 |
| **000510_hsa-miR-206_B** | 26.644 | Undetermined | 26.862 |
| **000528_hsa-miR-301_A** | 24.890 | Undetermined | 25.790 |
| **000546_hsa-miR-335_A** | Undetermined | 28.208 | 28.542 |
| **000565_hsa-miR-376a_A** | 28.601 | 28.461 | Undetermined |
| **000580_hsa-miR-20a_A** | 21.150 | Undetermined | 22.803 |
| **000583_hsa-miR-9_A** | 27.200 | Undetermined | 27.368 |
| **000592_hsa-miR-136_A** | 20.833 | Undetermined | 14.791 |
| **000602_hsa-miR-30b_A** | 17.279 | 20.996 | Undetermined |
| **001182_mmu-miR-124a_A** | 27.099 | Undetermined | 27.895 |
| **001187_mmu-miR-140_A** | 23.994 | Undetermined | 25.544 |
| **001271_hsa-miR-363_A** | 25.662 | Undetermined | 25.567 |
| **001515_hsa-miR-660_A** | 22.539 | Undetermined | 22.818 |
| **001518_hsa-miR-532_A** | 21.799 | Undetermined | 24.129 |
| **001550_hsa-miR-596_B** | 9.640 | 13.445 | Undetermined |
| **001587_hsa-miR-614_B** | Undetermined | 32.149 | 31.735 |
| **001613_hsa-miR-571_B** | 14.833 | 17.315 | Undetermined |
| **001614_hsa-miR-572_B** | 24.263 | Undetermined | 23.729 |
| **001621_hsa-miR-580_B** | Undetermined | 17.760 | 15.687 |
| **001984_hsa-miR-590-5p_A** | 24.474 | Undetermined | 17.341 |
| **001996_hsa-miR-454#_B** | 30.058 | Undetermined | 29.992 |
| **001998_hsa-miR-769-5p_B** | 26.641 | Undetermined | 26.066 |
| **002083_hsa-miR-502-3p_A** | 27.138 | 30.793 | Undetermined |
| **002087_hsa-miR-505#_B** | 26.206 | Undetermined | 28.239 |
| **002099_hsa-miR-224_A** | 25.752 | Undetermined | 24.727 |
| **002136_hsa-miR-33a#_B** | 27.284 | Undetermined | 28.224 |
| **002137_hsa-miR-92a-1#_B** | Undetermined | 30.572 | 34.151 |
| **002141_hsa-miR-99a#_B** | 28.486 | Undetermined | 27.054 |
| **002145_hsa-miR-141#_B** | 26.621 | Undetermined | 25.017 |
| **002158_hsa-miR-125b-2#_B** | 13.517 | Undetermined | 15.448 |
| **002159_hsa-miR-135b#_B** | 17.917 | Undetermined | 22.300 |
| **002160_hsa-miR-148b#_B** | 31.460 | Undetermined | 28.963 |
| **002231_hsa-miR-9#_B** | 33.483 | Undetermined | 31.053 |
| **002237_hsa-miR-548d-5p_A** | 28.952 | Undetermined | 28.789 |
| **002248_hsa-miR-142-5p_A** | 28.485 | Undetermined | 30.827 |
| **002254_hsa-miR-151-3p_B** | 5.793 | 6.907 | Undetermined |
| **002257_hsa-miR-339-5p_A** | 21.940 | Undetermined | 22.216 |
| **002258_hsa-miR-340_A** | 25.897 | Undetermined | 27.300 |
| **002259_hsa-miR-340#_B** | 26.792 | Undetermined | 27.926 |
| **002263_hsa-miR-190b_B** | Undetermined | 16.364 | 17.816 |
| **002268_hsa-miR-874_A** | 25.814 | Undetermined | 27.385 |
| **002271_hsa-miR-185_A** | 26.411 | Undetermined | 26.858 |
| **002272_hsa-miR-192#_B** | 32.455 | Undetermined | 32.703 |
| **002278_hsa-miR-145_A** | 28.698 | Undetermined | 32.038 |
| **002281_hsa-miR-193a-5p_A** | 23.972 | Undetermined | 24.571 |
| **002284_hsa-miR-138_A** | 24.008 | Undetermined | 28.456 |
| **002296_hsa-miR-885-5p_A** | 27.883 | Undetermined | 27.541 |
| **002297_hsa-miR-422a_A** | 29.658 | Undetermined | 29.205 |
| **002301_hsa-miR-22#_B** | 24.942 | Undetermined | 25.223 |
| **002304_hsa-miR-199a-3p_A** | 28.774 | Undetermined | 28.482 |
| **002322_hsa-miR-671-3p_A** | 28.873 | Undetermined | 28.657 |
| **002329_hsa-miR-452_A** | 23.835 | Undetermined | 26.143 |
| **002333_hsa-miR-181c#_B** | 25.060 | Undetermined | 23.863 |
| **002341_hsa-miR-708_A** | 26.674 | Undetermined | 27.826 |
| **002365_hsa-miR-494_A** | 24.739 | 29.544 | Undetermined |
| **002374_hsa-miR-887_A** | 28.161 | Undetermined | 27.775 |
| **002408_hsa-miR-548b-5p_A** | 28.765 | Undetermined | 28.702 |
| **002410_hsa-miR-550_B** | 28.771 | 28.914 | Undetermined |
| **002414_hsa-miR-616_A** | 26.808 | Undetermined | 26.408 |
| **002435_hsa-miR-501-3p_A** | 26.665 | Undetermined | 27.014 |
| **002437_hsa-miR-20a#_B** | 27.432 | Undetermined | 26.021 |
| **002444_hsa-miR-26b#_B** | Undetermined | 31.182 | 32.274 |
| **002658_HSA-MIR-338-5P_B** | 7.028 | Undetermined | 4.963 |
| **002677_HSA-MIR-590-3P_B** | 29.167 | Undetermined | 26.785 |
| **002779_HSA-MIR-1271_B** | 28.509 | Undetermined | 31.147 |
| **002791_HSA-MIR-1244_B** | 23.909 | Undetermined | 24.827 |
| **002822_HSA-MIR-1285_B** | 27.522 | Undetermined | 29.645 |
| **002838_HSA-MIR-1291_B** | 28.940 | Undetermined | 27.673 |
| **002840_HSA-MIR-1275_B** | 16.700 | Undetermined | 20.147 |
| **002841_HSA-MIR-1183_B** | Undetermined | 7.825 | 3.871 |
| **002843_HSA-MIR-1276_B** | 25.864 | Undetermined | 27.628 |
| **002852_HSA-MIR-1262_B** | 29.815 | Undetermined | 31.227 |
| **000382_hsa-let-7f_A** | 23.823 | Undetermined | Undetermined |
| **000389_hsa-miR-15a_A** | 25.743 | Undetermined | Undetermined |
| **000399_hsa-miR-23a_A** | Undetermined | Undetermined | 25.355 |
| **000427_hsa-miR-34b_B** | 31.773 | Undetermined | Undetermined |
| **000456_hsa-miR-130b_A** | 26.370 | Undetermined | Undetermined |
| **000489_hsa-miR-190_A** | 27.355 | Undetermined | Undetermined |
| **000516_hsa-miR-213_B** | Undetermined | Undetermined | 31.492 |
| **000524_hsa-miR-221_A** | 23.800 | Undetermined | Undetermined |
| **000527_hsa-miR-296_A** | 27.242 | Undetermined | Undetermined |
| **000533_hsa-miR-302c_A** | Undetermined | Undetermined | 16.695 |
| **000539_hsa-miR-324-5p_A** | 28.386 | Undetermined | Undetermined |
| **000554_hsa-miR-361_A** | 26.199 | Undetermined | Undetermined |
| **000555_hsa-miR-367_A** | Undetermined | Undetermined | 17.754 |
| **000567_hsa-miR-378_B** | Undetermined | 27.829 | Undetermined |
| **001037_hsa-miR-490_A** | Undetermined | Undetermined | 22.426 |
| **001047_hsa-miR-501_A** | 26.915 | Undetermined | Undetermined |
| **001052_hsa-miR-508_A** | 27.017 | Undetermined | Undetermined |
| **001109_hsa-miR-502_A** | 28.055 | Undetermined | Undetermined |
| **001111_hsa-miR-511_A** | 27.778 | Undetermined | Undetermined |
| **001116_hsa-miR-520b_A** | Undetermined | Undetermined | 28.605 |
| **001119_hsa-miR-520e_A** | Undetermined | Undetermined | 24.808 |
| **001153_hsa-miR-517c_A** | 26.750 | Undetermined | Undetermined |
| **001156_hsa-miR-518b_A** | Undetermined | Undetermined | 30.211 |
| **001193_mmu-miR-187_A** | Undetermined | Undetermined | 22.954 |
| **001280_hsa-miR-455_A** | 31.323 | Undetermined | Undetermined |
| **001286_hsa-miR-539_A** | 29.893 | Undetermined | Undetermined |
| **001522_hsa-miR-554_B** | 30.961 | Undetermined | Undetermined |
| **001543_hsa-miR-589_B** | 25.128 | Undetermined | Undetermined |
| **001560_hsa-miR-627_A** | Undetermined | Undetermined | 20.315 |
| **001582_hsa-miR-638_B** | Undetermined | Undetermined | 27.868 |
| **001591_hsa-miR-617_B** | 26.605 | Undetermined | Undetermined |
| **001593_hsa-miR-618_A** | Undetermined | Undetermined | 19.567 |
| **001599_hsa-miR-646_B** | Undetermined | Undetermined | 13.357 |
| **001606_hsa-miR-661_B** | Undetermined | Undetermined | 21.428 |
| **001607_hsa-miR-662_B** | Undetermined | Undetermined | 14.135 |
| **001608_hsa-miR-449b_A** | 24.667 | Undetermined | Undetermined |
| **001615_hsa-miR-573_B** | Undetermined | 20.738 | Undetermined |
| **001823_hsa-miR-512-3p_A** | 23.803 | Undetermined | Undetermined |
| **002093_hsa-miR-486-3p_A** | 21.495 | Undetermined | Undetermined |
| **002105_hsa-miR-186#_B** | 31.976 | Undetermined | Undetermined |
| **002116_hsa-miR-361-3p_B** | Undetermined | Undetermined | 27.207 |
| **002152_hsa-miR-922_B** | Undetermined | Undetermined | 30.724 |
| **002166_hsa-miR-29b-2#_B** | 27.074 | Undetermined | Undetermined |
| **002177_hsa-miR-934_B** | Undetermined | Undetermined | 32.927 |
| **002181_hsa-miR-938_B** | 34.664 | Undetermined | Undetermined |
| **002203_hsa-miR-875-5p_B** | Undetermined | 11.558 | Undetermined |
| **002215_hsa-miR-196b_A** | 29.849 | Undetermined | Undetermined |
| **002216_hsa-miR-128a_A** | 27.096 | Undetermined | Undetermined |
| **002217_hsa-miR-18b_A** | 28.507 | Undetermined | Undetermined |
| **002245_hsa-miR-122_A** | Undetermined | Undetermined | 27.608 |
| **002249_hsa-miR-143_A** | 27.530 | Undetermined | Undetermined |
| **002253_hsa-miR-101_A** | Undetermined | 28.774 | Undetermined |
| **002262_hsa-miR-147b_A** | 26.813 | Undetermined | Undetermined |
| **002267_hsa-miR-545_A** | 32.025 | Undetermined | Undetermined |
| **002274_hsa-miR-200b#_B** | Undetermined | Undetermined | 18.963 |
| **002289_hsa-miR-139-5p_A** | 27.750 | Undetermined | Undetermined |
| **002306_hsa-miR-214_A** | 25.406 | Undetermined | Undetermined |
| **002307_hsa-let-7a#_B** | Undetermined | Undetermined | 8.565 |
| **002325_hsa-miR-744#_B** | Undetermined | Undetermined | 26.534 |
| **002339_hsa-miR-483-3p_B** | Undetermined | 27.273 | Undetermined |
| **002352_hsa-miR-652_A** | 21.988 | Undetermined | Undetermined |
| **002384_hsa-miR-519b-3p_B** | Undetermined | 6.992 | Undetermined |
| **002402_hsa-miR-517a_A** | 29.027 | Undetermined | Undetermined |
| **002419_hsa-miR-15a#_B** | Undetermined | Undetermined | 28.456 |
| **002420_hsa-miR-16-1#_B** | 24.632 | Undetermined | Undetermined |
| **002422_hsa-miR-18a_A** | 29.778 | Undetermined | Undetermined |
| **002428_hsa-miR-500_A** | 26.521 | Undetermined | Undetermined |
| **002429_hsa-miR-548c-5p_A** | Undetermined | Undetermined | 30.836 |
| **002431_hsa-miR-625_A** | 29.910 | Undetermined | Undetermined |
| **002441_hsa-miR-24-2#_B** | Undetermined | Undetermined | 25.337 |
| **002447_hsa-miR-29a#_B** | 26.537 | Undetermined | Undetermined |
| **002678_HSA-MIR-191#_B** | 33.379 | Undetermined | Undetermined |
| **002761_HSA-MIR-1236_B** | Undetermined | Undetermined | 26.673 |
| **002776_HSA-MIR-1179_B** | 33.740 | Undetermined | Undetermined |
| **002781_HSA-MIR-1201_B** | 29.238 | Undetermined | Undetermined |
| **002792_HSA-MIR-1303_B** | Undetermined | Undetermined | 26.835 |
| **002807_HSA-MIR-1270_B** | Undetermined | Undetermined | 27.657 |
| **002818_HSA-MIR-1254_B** | 28.371 | Undetermined | Undetermined |
| **002847_HSA-MIR-1180_B** | 27.521 | Undetermined | Undetermined |
| **002850_HSA-MIR-1256_B** | 34.883 | Undetermined | Undetermined |
| **002868_HSA-MIR-1249_B** | Undetermined | Undetermined | 33.519 |
| **002885_HSA-MIR-1267_B** | Undetermined | 25.652 | Undetermined |
| **002893_HSA-MIR-1247_B** | Undetermined | Undetermined | 21.394 |
| **002909_HSA-MIR-548I_B** | Undetermined | Undetermined | 34.193 |
